# Knowledge, attitudes and practices of smallholder dairy farmers on antimicrobial use in selected districts of Zambia: implications for antimicrobial stewardship

**DOI:** 10.3389/fvets.2026.1763931

**Published:** 2026-06-11

**Authors:** Lweendo Hachamba, Ethel M'kandawire, John Bwalya Muma, Flavien Bumbangi, Noela Lukwesa, Mapeesho Kamayani, Merning Mwenifumbo, Mette Helen Bjorge Müller

**Affiliations:** 1 Department of Environmental Health, School of Medicine, Eden University, Barlastone Park, Lusaka, Zambia; 2 Department of Disease Control, School of Veterinary Medicine, University of Zambia, Lusaka, Zambia; 3 School of Medicine, Eden University, Barlastone Park, Lusaka, Zambia; 4 Churches Health Association of Zambia, Lusaka, Zambia; 5 Department of Veterinary Public Health and Epidemiology, Lilongwe University of Agriculture and Natural Resources, Lilongwe, Malawi; 6 Faculty of Veterinary Medicine, Norwegian University of Life Sciences, NMBU, Ås, Norway

**Keywords:** antibiotic resistance, antibiotic stewardship, antibiotic use, antibiotics, attitudes, knowledge, practices, smallholder dairy farmers

## Abstract

**Background:**

The improper use of antibiotics in dairy farming contributes significantly to the development of antibiotic resistance (AMR), posing a global public health risk. Antimicrobial stewardship (AMS) in dairy production can reduce the spread of AMR. Smallholder dairy farmers (SHDFs) in low- and middle-income countries (LMICs) are particularly critical stakeholders in this context, given their significant contribution to the dairy sector. Consequently, AMS in dairy production can reduce the spread of AMR. This study aimed to examine the knowledge, attitudes, and practices (KAP) related to antibiotic use (AMU) and AMR among SHDFs in selected districts of Zambia to guide AMS interventions in the given contexts.

**Methods:**

A cross-sectional survey was conducted using a structured questionnaire administered to 360 SHDFs conveniently identified at Milk Collection Centers (MCCs) across six purposively selected districts of Southern and Lusaka provinces.

**Results:**

In total, 75.8% of respondents had poor overall KAP scores. While attitudes were positive toward the need to consult a veterinarian (99.2%), some practices were misaligned, including self-administration (38.6%), failure to observe withdrawal periods (24.2%) and selling (20%) and consuming milk (19.7%) from drug-treated animals. Whereas 42% could define AMR only 25.6% of participants knew the effects of misusing antibiotics. Good KAP was associated with level of education (*p* < 0.001) and geographic location (*p* < 0.001). Districts with a more urban setup in their respective provinces had higher overall KAP scores compared to those that were not (Monze District of Southern Province and Chilanga District of Lusaka Province). Those who had received prior training in appropriate antibiotic use and animal health management had higher odds of having good KAP (*p* < 0.036).

**Conclusion:**

This study gave insights into the KAP among SHDFs regarding AMU and AMR with implications for AMS. Educational programmes that shape correct attitudes based on increased Knowledge are encouraged. Geographical disparities in KAP scores emphasize the need for targeted context-specific AMS interventions. Our findings also revealed that financial barriers undermined prudent antimicrobial use. Addressing these gaps through training, economic support, and district-focused One Health interventions is essential to strengthening AMS in Zambia.

## Introduction

Antimicrobial resistance (AMR) is widely regarded as one of the most pressing global health threats of the 21st century. The World Health Organization (WHO) describes it as a “silent pandemic” threatening human health, food safety, and economic stability (1). In 2019 alone, AMR was associated with an estimated 4.95 million deaths globally, with the highest burden recorded in sub-Saharan Africa and other low- and middle-income countries (LMICs) (2). This burden is projected to rise to 10 million annual deaths by 2050 if no effective action is taken (1).

Extensive antimicrobial use (AMU) in food-producing animals is recognized as the main driver of AMR in humans (3, 4). The livestock sector including dairy farming has come under increasing scrutiny for its role in promoting the development and spread of antibiotic residues (ARs) and resistant pathogens through the food chain and environment (5). In both high-income countries and LMICs, antibiotics are used therapeutically, prophylactically, and at times non-therapeutically to promote growth (6). While such use can increase productivity, the improper or excessive use of antibiotics raises significant concerns for food safety and public health. Common misuse practices include; using antibiotics to treat viral infections, prevent non-infectious disease in healthy animals, promote growth, and administering incorrect dosages or durations of treatment (5, 7). Additionally, failure to comply with withdrawal periods and inappropriate prescribing practices contribute to the persistence of residues in milk and other animal products, increasing the risk of resistant bacterial strains entering the human food chain (1, 8).

In LMICs, particularly across Africa, misuse of antibiotics in livestock production is widely reported (9, 10). In many regions, antibiotics are readily accessible without veterinary prescription, often obtained through informal channels (2). KAP studies in various regions of Africa have demonstrated critical gaps. These include low awareness of AMR, reliance on antibiotics as first-line treatment even for non-bacterial infections and poor milk disposal practices when antibiotics are used, self-prescription, incorrect dosages, and non-compliance with withdrawal periods as persistent challenges (11–13). Poor KAP among farmers is worsened by systemic factors, such as limited veterinary access, weak or lack of regulations, limited diagnostic capacity, and economic constraints (11, 14). These factors contribute to the accumulation of ARs in milk and the emergence of resistant strains. Several studies across Africa have reported the presence of ARs and antibiotic-resistant pathogens in raw milk, raising serious concerns about food safety and public health (15, 16). Residues of commonly used antibiotics such as tetracyclines, sulfonamides, and penicillins have been detected in milk samples from countries including Zimbabwe, Tanzania, Kenya, and Niger, often exceeding acceptable limits due to non-compliance with withdrawal periods (12, 17–19). In addition, multidrug-resistant bacteria such as *Escherichia coli, Staphylococcus aureus*, and *Salmonella spp*. have been isolated from raw milk, some carrying resistance genes against beta-lactams and other critical antimicrobials (20–22). These findings highlight the need to promote antimicrobial stewardship (AMS) at the farm level. AMS refers to a set of coordinated strategies aimed at optimizing antimicrobial use, minimizing resistance, and reducing harm to human and animal health (8). AMS educational campaigns have not been widely developed in the agricultural sector of LMICs and have mainly focused on human medicine (23). Effective AMS in livestock systems requires not only policy enforcement but also behavioral change guided by a sound understanding of farmers' knowledge, attitudes, and practices (KAP).

In Zambia, Smallholder dairy farmers (SHDFs) who contribute a substantial proportion of milk production, often exceeding 80% of total milk supply take their milk to milk collection centers (MCCs) (24, 25). Local studies have confirmed the presence of ARs and resistant genes in raw milk supplied by SHDFs. Muma and others (26) reported a 30.12% prevalence of antibiotic residues in milk samples from Lusaka Province. More recently, Mwasinga and others (27) identified multidrug-resistant *Escherichia coli* in milk collected from MCCs in Namwala District of Southern province. Despite this evidence, a lack of comprehensive data on the KAP of SHDFs in Zambia remains. Understanding the behavioral and systemic drivers of AMU is essential for designing AMS interventions to reduce the cases of AMR and improve overall food safety of animal-derived foods. The KAP model helps identify gaps in KAP that can inform targeted, context-specific interventions. Therefore, this study aimed to document KAP related to AMU and AMR among SHDFs in selected districts of Zambia. In addition, this study will contribute to evidence-based recommendations for promoting AMS among SHDFs in Zambia.

## Materials and methods

### Study design, study area, and study population

**Figure 1:**
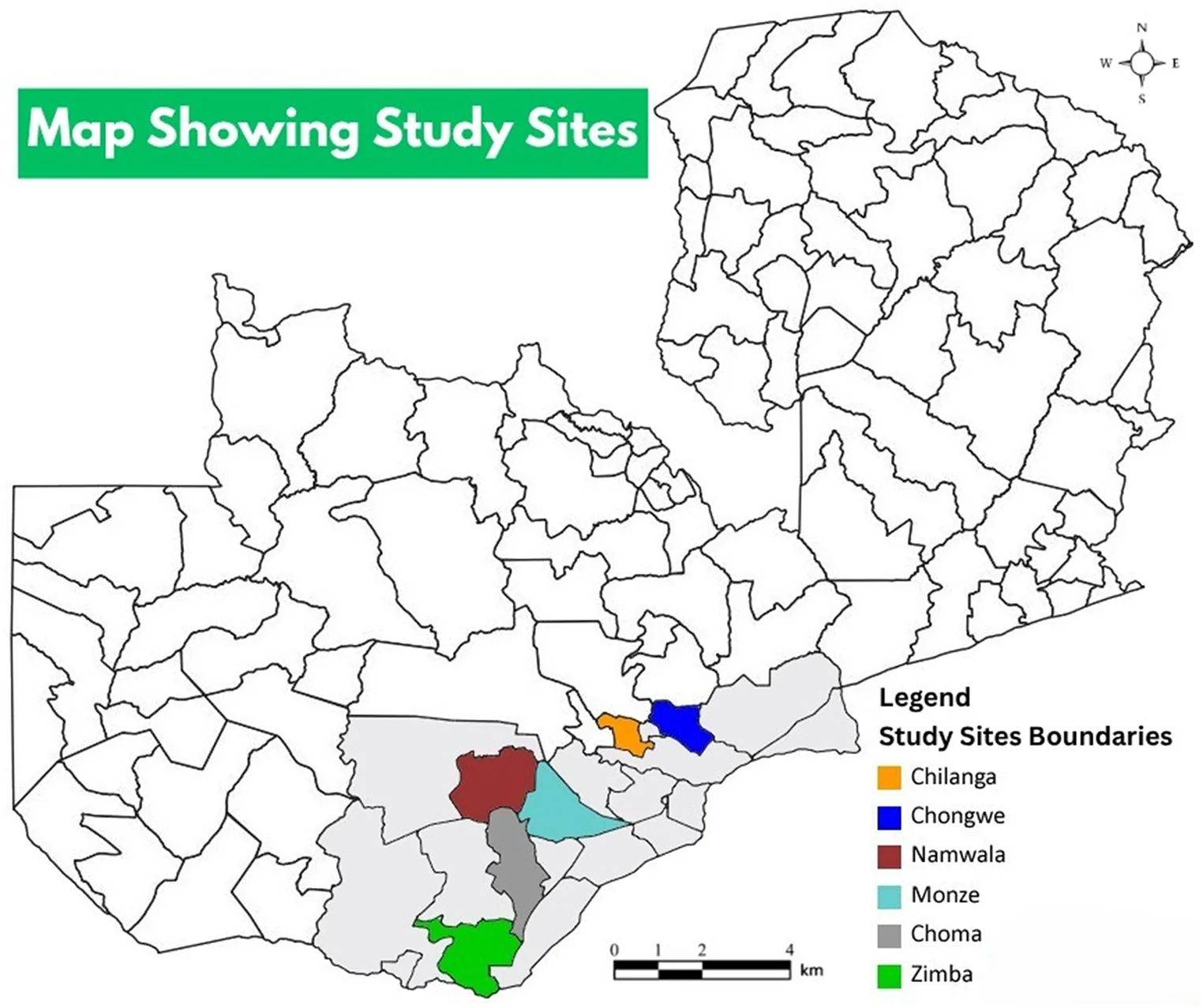
Map of study sites.

A cross-sectional study was undertaken in the Southern and Lusaka provinces of Zambia from August to September 2025 (Figure 1). In the Southern province, we purposively sampled four districts: Choma, Monze, Namwala, and Zimba. In the Lusaka province, we purposively selected two districts: Chilanga and Chongwe. These districts are known for having significant small-scale milk production in their respective provinces.

The target population consisted of smallholder dairy farmers who are members of their local Dairy Cooperatives, both male and female, aged 18 years and above. Smallholder dairy farmers were considered small-scale producers who depend on family labour and produce milk for both home consumption and market sale, with an average herd size of 4 (25, 28). SHDFs were selected as they contribute a substantial proportion of milk in the country (24, 25).

### Sample size estimation and sampling procedure

Sample size estimation was conducted using Epitools (http://epitools.ausvet.com.au/). With an estimated 5,408 dairy farmers affiliated with their respective cooperatives in both provinces, the sample size was determined based on an assumption of 50% prevalence of ‘good' KAP and 5% desired precision at a 95% confidence level. In the absence of prior data on the proportion of good knowledge in this population, a prevalence of 50 percent was assumed to maximize sample size (1). A sample size of 360 was estimated. The Southern province had 5,091 registered farmers, and Lusaka province had 317, resulting in a proportional distribution of 339 to 21 respondents from each province, respectively. With a small sample size from Lusaka province, statistical power to analyse KAP would have been inadequate. To overcome this limitation, the sample distribution was adjusted to 250 and 110, respectively, maintaining the dominance of the Southern province. This adjustment reflected an adequate representation from Lusaka province. Similar sampling adjustments have been applied in other KAP studies where oversampling of participants was necessary to strengthen statistical analyses (11, 28). The number of farmers interviewed was proportionate to the quantity of bulk milk supply per district at the time, ensuring equitable representation. Convenient sampling was employed, and participants were engaged voluntarily at the MCCs with the help of the MCC managers. They were assured of anonymity and allowed to leave at any time during the interview if they wished to.

### Data collection

The questionnaire design was informed by previous studies (11, 29). The structured questionnaire was administered during face-to-face interviews to all eligible dairy farmers. The questionnaires were originally written in the English language and translated into the local language (Tonga) of the Southern Province for participants who were unable to respond in English and (Nyanja) for those in Lusaka province. Translation accuracy was ensured through forward translation into local languages (Tonga and Nyanja) followed by back-translation into English.

A pilot study was conducted among 36 randomly selected farmers to pretest the questionnaire for face and content validity, and the questionnaire was adjusted accordingly. The selection of several farmers for pre-testing was based on 10% of the study's sample size. Internal consistency reliability was assessed using Cronbach's alpha for each KAP domain which gave alpha (>0.9). The findings from the pilot study were not included in the analysis of the main research. A total of 43 questions on KAP were administered. Most of the questions were multiple-choice and were classified as either correct or incorrect. Answers to open-ended questions were coded into categorical variables. The questionnaire consisted of four sections. Section A (3 questions) focused on the socio-demographic information of the participants; Section B (13 questions) addressed knowledge regarding the use of antibiotics in dairy production; Section C (8 questions) included questions related to the participants' attitudes (perceptions about antibiotic misuse), and Section D (18 questions) covered practices (antibiotic usage and withdrawal periods) concerning the use of antibiotics in their livestock. In Section D, 11 questions were used to assess the practices, while the remaining questions, such as the types of antibiotics used, provided contextual insights into farmer practices in this study area. Therefore, the KAP was assessed based on 32 questions.

### Data analysis

After collecting the data, data entry was performed using Microsoft Excel version 16.22, and the data were further analyzed using RStudio version 4.4. To determine KAP scores, the method described in (30) was adopted. One mark was awarded for each correct answer, while zero was given for incorrect or uncertain responses. Multiple-response questions were scored using a structured system that awarded one point for exclusively correct or predominantly correct answers, while responses that included incorrect options received no score. This method ensured an accurate reflection of respondents' KAP without inflating scores due to guessing. For KAP studies, Blooms criteria categorize scores of 80%−100% as good, 60%−79% as moderate, and < 60% as poor (2, 3). In this study, we used a cutoff of 75%, which was a modification of the Blooms cutoff point. Similar peer-reviewed studies have adopted a dichotomized classification using a 75% threshold to define good scores (4, 5). Therefore, a cut-off point of 75% was established to define the KAP scores as “good” or “poor”. Participants with ≥75% correct responses in the knowledge, attitude or practice questions were categorized as having good knowledge, good attitude and good practices, respectively. On the other hand, those scoring < 75% were classified as having poor knowledge, poor attitude and poor practices. In addition, the percentage of correct answers for each participant was calculated for all 32 KAP questions. Respondents having ≥75% correct answers in all the knowledge, attitude and practice questions were considered as having good KAP scores and those with < 75% were considered as having poor KAP scores.

To identify the normal distribution of the KAP score, the Shapiro–Wilk normality test was applied. Since the scores were not normally distributed (*W* = 0.949, *p* < 0.001), Spearman's rank correlation was employed to explore the correlation values between the outcomes of Knowledge, Attitude, and Practice. Chi-square tests were used for the association between demographics and KAP levels. Logistic Regression was performed to identify the predictors of good KAP outcomes. Independent variables, including age group, gender, education level, and district, were included in this model. The KAP levels of “good” vs. “poor” were the dependent variable in the model. The demographic factors that showed a significant association (*p* < 0.25) during the chi-square univariate analysis were used to perform a multivariable binary logistic regression analysis to identify the key independent variables affecting KAP toward AMU and AMR in the study area.

## Results

### Demographic characteristics of the study population

A total of 360 farmers participated in the study. Southern province had 250 (69%) respondents while Lusaka had 110 (31%). Most of the participants were male (84%). The age group which had most participants was 35–44 years (31.4%). The highest level of education for most of the participants was primary education (40%) (Table 1).

**Table 1:** Summary of demographic characteristics.

Variable	Category	Frequency
Sex	Male	303 (84%)
	Female	57 (16%)
Age	18–24	17 (4.7%)
	25–34	82 (22.7%)
	35–44	113 (31.4%)
	45–54	83 23.1%)
	55–64	37 (10.3%)
	65+	28 7.8%)
Formal education	Primary	143 (40%)
	Secondary	110 (31%)
	Tertiary	95 (26%)
	No Education	12 (3%)
District	Chilanga	36 (10%)
	Choma	65 (18.1%)
	Chongwe	74 (20.6%)
	Monze	85 (13.9%)
	Namwala	50 (13.9%)
	Zimba	50 (13.9%)
Province	Southern	250 (69.4%)
	Lusaka	110 (30.6%)

### Knowledge

**Figure 2:**
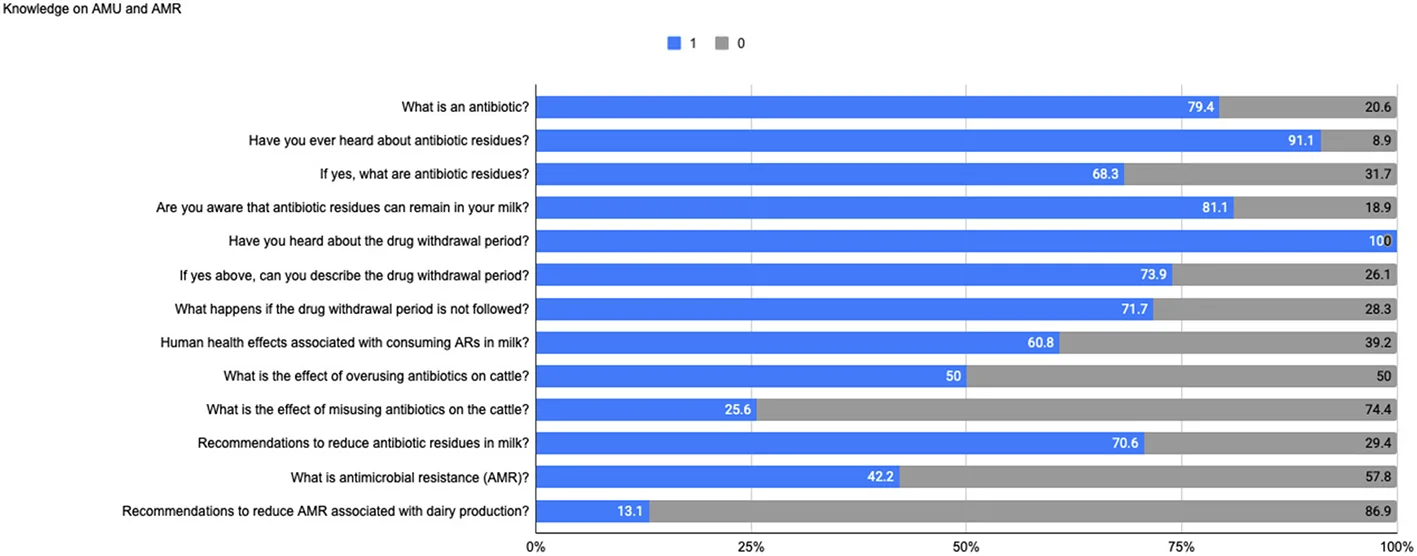
Knowledge on AMU and AMR.

Knowledge was assessed against 11 items (Figure 2). A significant proportion of participants (79.4%; 286/360) were able to define an antibiotic correctly, however only 25.6% (92/360) of participants knew the effects of misusing antibiotics with regard to dosage or number of treatments. About 42% (152/360) could accurately define AMR.

Overall, 58.6% of respondents demonstrated good knowledge. Individual scores ranged from 16.7 to 100%. Knowledge differed significantly by age, education, and district (Supplementary Table S1). Farmers aged 45–54 (71.1%) and 55–64 (73%) were more likely to have good knowledge compared to younger groups (*p* = 0.022). Education showed a clear gradient, with tertiary-educated farmers achieving the highest knowledge scores (71.6%) compared to only 41.7% among those with no formal education (*p* < 0.001). District-level variation was also significant (*p* < 0.001): Monze (91.8%) and Zimba (84%) recorded the highest proportions with good knowledge, while Chongwe (27%) and Chilanga (25%) had the lowest.

### Attitudes

**Figure 3:**
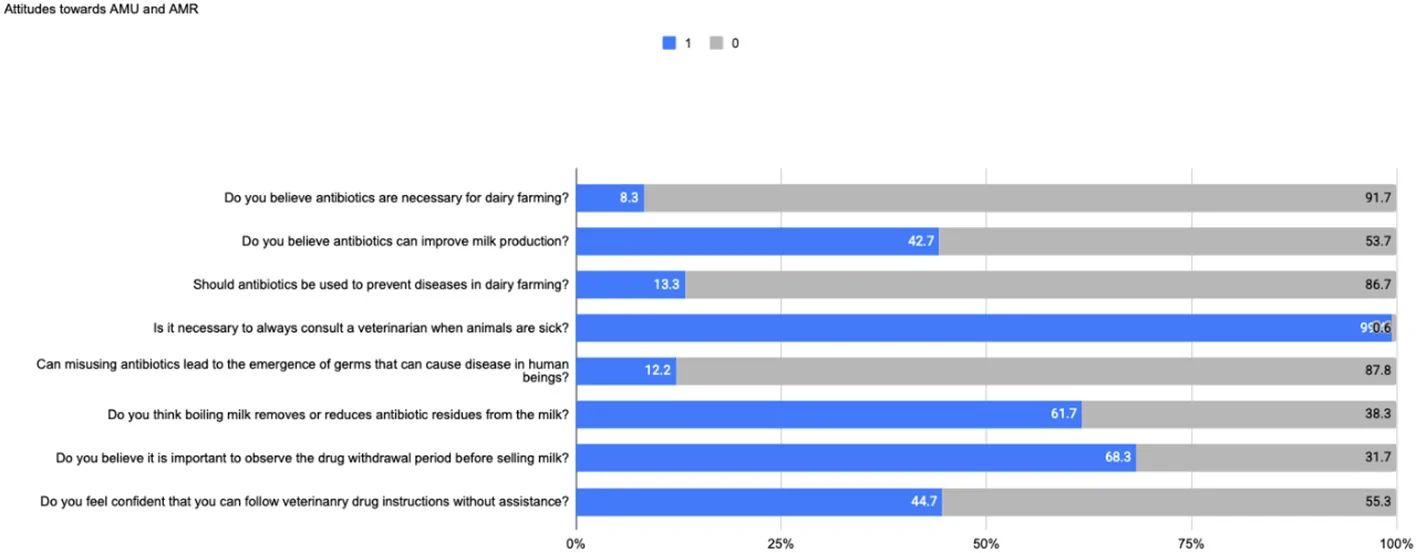
Attitudes towards AMU and AMR.

Attitudes were assessed against 8 items (Figure 3). A large proportion (86.7%; 312/360) of farmers believed that antibiotics should be used for disease prevention. Interestingly, 38.3% (144/360) thought that boiling milk could remove or reduce antibiotic residues from the milk.

Overall, only 14.4% of farmers demonstrated good attitudes. Individual attitude scores ranged from 12.5 to 100%. Females (26.3%) expressed more favorable attitudes compared to males (12.2%) (*p* = 0.005) (Supplementary Table S2). Attitudes improved with higher education: 27.4% of tertiary respondents had good attitudes vs. 8.3% among those with no formal education (*p* < 0.001). Older farmers (65+, 32.1%) were also more likely to hold favorable attitudes than younger groups (*p* = 0.012). District-level differences were evident, with Chilanga (33%) showing the highest positive attitudes compared to Chongwe (6.8%) (*p* = 0.006).

### Practices

**Figure 4:**
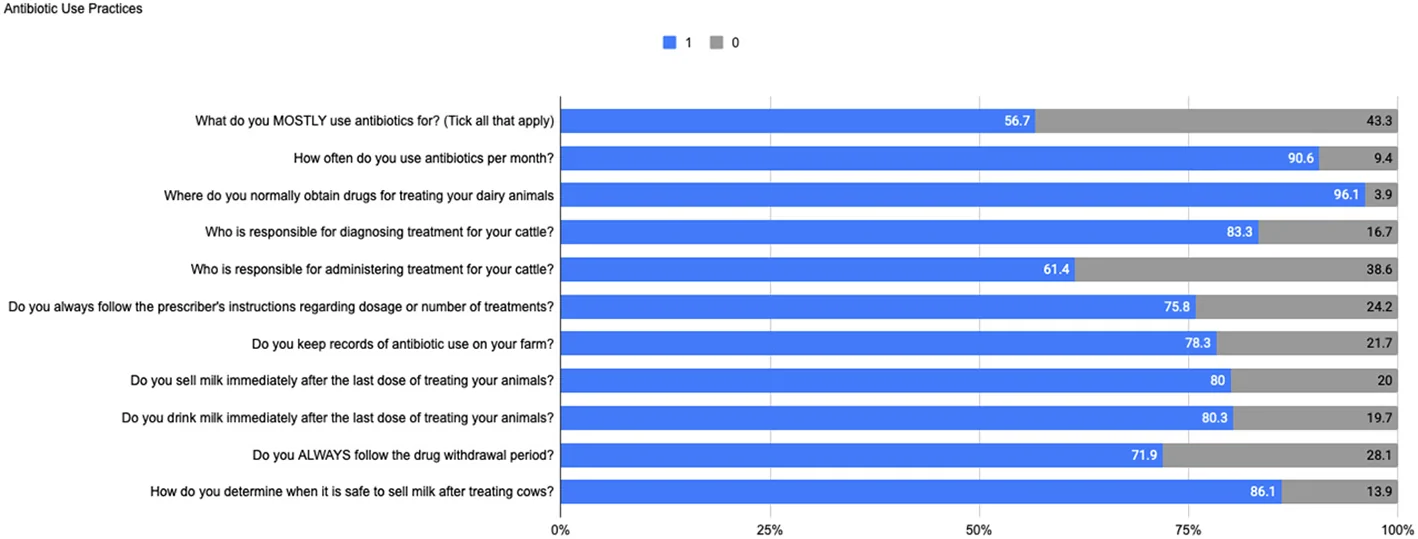
Antibiotic use practices.

**Figure 5:**
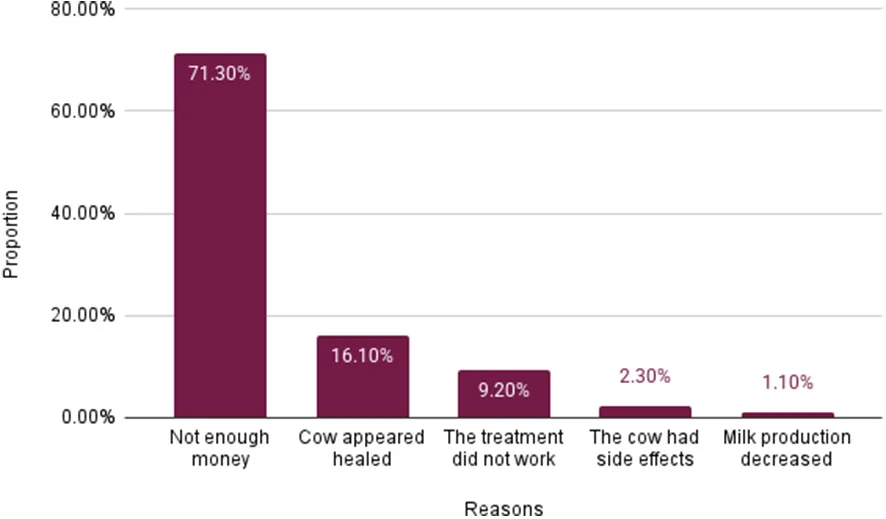
Reasons for not following prescription.

Practices were assessed against 11 items (Figure 4). All farmers indicated that they used antibiotics. The most used classes of antibiotics reported in the study areas were tetracyclines, penicillins, and sulfonamides. About forty percent (43.3%; 156/360) of SHDFs reported using antibiotics for disease prevention and/or growth promotion, in addition to treating disease. About a quarter (24.2%; 87/360) of the study population revealed that they do not always follow the prescriber's instructions regarding dosage or number of treatments. The reasons cited for this poor practice were: not enough money (71.3%), the cow appeared healed (16.1%), the treatment did not work (16.1%) and the milk production decreased (1%) (Figure 5).

Overall, 62.2% of respondents reported good practices. Individual practice scores ranged from 9.1 to 100%. Educational attainment was significantly associated with practices (*p* = 0.011), with those at primary (69.9%) and tertiary (65.3%) levels showing better practices compared to those without education (41.7%) (Supplementary Table S3). Practices varied sharply by district (*p* < 0.001). Monze (92.9%) and Choma (89.2%) reported the highest adherence to good practices, whereas Namwala (14%) and Chongwe (32.4%) were relatively low.

### Multivariable analysis of KAP domains

Multivariable logistic regression identified district and education as the strongest predictors of good KAP outcomes (Table 2). Compared to Chilanga, farmers in Monze had 52.6 higher odds of good knowledge (95% CI: 16.1–171.5), and 11.3 higher odds of good practices (95% CI: 3.7–33.9). Farmers in Namwala had significantly lower odds of good practices (OR: 0.137; 95% CI: 0.046–0.414). Tertiary education was associated with higher odds of good attitudes (OR: 4.277) and practices (OR: 4.106), relative to no education.

**Table 2:** Multivariable logistic regression analysis of knowledge, attitudes and practices.

Variable	Category	Knowledge		Attitudes		Practices	
		OR	*p*- value	OR	*p*- value	OR	*p*-value
Gender	Male	–	–	0.647 (0.294–1.421)	0.285	–	–
	Female	Ref	–	Ref	–	Ref	–
Age Range	25–34	0.715 (0.205–2.49)	0.224	0.916 (0.097–8.637)	0.204	1.215 (0.285–5.177)	0.436
	35–44	0.577 (0.168–1.99)		2.880 (0.348–23.839)		1.267 (0.312–5.136)	
	45–54	1.449 (0.396–5.31)		1.609 (0.181–14.239)		2.145 (0.506–9.086)	
	55–64	1.356 (0.301–6.10)		2.035 (0.207–19.961)		2.643 (0.544–12.832)	
	65 +	1.010 (0.216–4.71)		3.542 (0.360–34.758)		2.749 (0.543–13.923)	
	18–24	Ref		Ref		Ref	
Education	Primary	0.692 (0.171–2.79)	0.004	1.202 (0.133–10.818)	0.024	2.822 (0.530–15.016)	0.171
	Secondary	1.776 (0.434–7.25)		1.750 (0.193–15.867)		1.973 (0.359–10.874)	
	Tertiary	2.396 (0.570–10.07)		4.277 (0.476–38.459)		4.106 (0.728–23.162)	
	No education	Ref		Ref		Ref	
District	Choma	5.376 (1.861–15.53)	< 0.001	0.443 (0.137–1.43)	0.032	8.052 (2.650–24.469)	< 0.001
	Chongwe	1.401 (0.528–3.72)		0.147 (0.043–0.495)		0.350 (0.147–0.828)	
	Monze	52.62 (16.146–171.54)		0.452 (0.169–1207)		11.250 (3.737–33.872)	
	Namwala	6.254 (2.1641–18.07)		0.227 (0.063–0.811)		0.137 (0.046–0.414)	
	Zimba	18.117 (5.658–58.01)		0.361 (0.119–1.098)		1.148 (0.437–3.020)	
	Chilanga	Ref		Ref		Ref	

Nagelkerke R^2^N: Knowledge (0.427), Attitudes (0.185), Practices (0.484).

### Overall KAP

Overall KAP scores were obtained by dividing by the total number of questions (30) by the total number of correct answers, expressed as percentages for everyone. The overall KAP scores were poor, as 75.8% (273/360) of participants scored below the cut-off point (Table 3). Gender was not statistically associated with KAP outcomes (*p* = 0.680) (Table 3). Similarly, no significant association was found with age (*p* = 0.710). A trend toward improved outcomes was observed with increasing education (*p* = 0.008). There were statistically significant differences in KAP outcomes by district (*p* < 0.001). When aggregated by province, Southern province had a higher prevalence of good KAP (29%) compared to Lusaka (11%). In Southern Province, Monze district recorded the highest KAP scores (61%), and in Lusaka province, it was Chilanga district (25%).

**Table 3:** Distribution of overall KAP scores across demographic variables.

Variable	Number	KAP outcome		*p*-value
		Good KAP	Poor KAP	
Gender				
Male	303	72(23.8%)	231(76.2%)	0.680
Female	57	15(26.3%)	42(73.7%)	
Age Range				
18–24	17	4 (23.5%)	13 (76.5%)	0.710
25–34	82	15 (18.3%)	67 (81.7%)	
35–44	113	27 (23.9%)	86(76.1%)	
45–54	83	22 (26.5%)	61 (73.5%)	
55–64	37	10 (27%)	27 (73%)	
65+	28	9 (32%)	19 (68%)	
Education				
No education	12	1 (8.3%)	11 (91.7%)	0.008
Primary	143	23 (16.1%)	120 (83.9%)	
Secondary	110	32 (29%)	78 (71%)	
Tertiary	95	31 (32.6%)	64 (67.4%)	
District				
Choma	65	16 (24.6%)	49 (75.4%)	< 0.001
Chongwe	74	3 (4%)	71 (96%)	
Monze	85	53 (62.4%)	32 (37.6%)	
Namwala	50	1 (2%)	49 (98%)	
Zimba	50	5 (10%)	45 (90%)	
Chilanga	36	9 (25%)	27 (75%)	
		**87**	**273**	

Multivariable logistic regression (Table 4) confirmed that both education and district were significant predictors of overall KAP (*p* < 0.001). Tertiary education increased the likelihood of achieving good outcomes by more than ten (OR: 10.588; 95% CI: 1.082–103.523). District differences persisted: Monze (OR: 6.899; 95% CI: 2.645–17.995) was most favorable, while Namwala (OR: 0.046; 95% CI: 0.005–0.394) was least likely to achieve good KAP. The model demonstrated good explanatory power (Nagelkerke *R*^2^ = 0.529).

**Table 4:** Logistic model for overall KAP.

Variable	OR (95% CI)	*p*-value
Education		
Primary	1.457 (0.156–13.574)	< 0.001
Secondary	4.379 (0.459–41.794)	
Tertiary	10.588 (1.082–103.523)	
No education	Ref	
District		
Choma	1.897 (0.665–5.411)	< 0.001
Chongwe	0.126 (0.031–0.522)	
Monze	6.899 (2.645–17.995)	
Namwala	0.0461 (0.005–0.394)	
Zimba	0.263 (0.0742–0.933)	
Chilanga	Ref	

Nagelkerke R^2^N: 0.534.

### Training and KAP outcome

Training on antimicrobial use was reported by 45.3% of farmers (Table 5). Chi-square analysis revealed a significant association between training and good KAP (χ^2^ = 5.65, *p* = 0.017) (Supplementary Table S4). Farmers who had received training in appropriate antibiotic use and animal health management were nearly twice as likely to achieve good KAP compared to those who had not (OR: 0.556; 95% CI: 0.342–0.905).

**Table 5:** Contingency table of KAP outcome and training.

KAP Outcome			
Training	Good	Poor	Total
No	38	159	197
Yes	49	114	163
Total	87	273	360

### Correlation between knowledge, attitude, and practice

Spearman's rank correlation showed significant positive relationships among the three KAP domains (Table 6). Knowledge was positively correlated with attitudes (*r* = 0.165, *p* = 0.002) and practices (*r* = 0.306, *p* < 0.001). Attitudes were also positively correlated with practices (*r* = 0.281, *p* < 0.001). The strongest association was between knowledge and practices.

**Table 6:** Spearman rank correlation coefficients between the KAP scores.

Variables	Correlation coefficients	*p*–value
Knowledge–attitude	0.165	0.002
Knowledge–practices	0.306	< 0.001
Attitude- practices	0.281	< 0.001

## Discussion

AMR has emerged as one of the greatest threats to human, animal, and environmental health, with misuse of antibiotics in livestock production consistently identified as a key driver (1). Understanding the knowledge, attitudes, and practices (KAP) of smallholder dairy farmers is therefore not merely descriptive, but fundamental for designing interventions that promote AMS to safeguard food safety and public health. The findings of the present study provided some critical insights into the KAP of SHDFs in select districts of Zambia and implications on AMS.

### Knowledge

While majority of respondents (79.4%) correctly defined antibiotics and an even higher proportion (91.1%) had heard of ARs, only 68.3% could accurately describe what ARs are. This disconnect between awareness and accurate understanding of ARs is not unique to Zambia and has been documented in other LMIC contexts. For instance, Caudell and others (11) reported that although 84% of livestock keepers in Tanzania had heard of antibiotic residues, less than half could describe their health implications or the importance of observing drug withdrawal periods. Similarly, a study by Kimera and others (29) found that only 61% of cattle farmers in Northern Tanzania knew that residues could remain in milk post-treatment, and fewer understood the risks to consumers, including allergic reactions and resistance selection.

Notably, only 42.2% could correctly define AMR, and 13.1% understood how AMR could be mitigated in the dairy sector. These figures indicate that while general awareness is high, there is limited depth of understanding of the critical AMR principles. These findings align with similar studies in Nigeria, Ethiopia, Namibia and Cameroon where poor knowledge of resistance development and public health risks associated with misuse of antibiotics were indicated (13, 30–32).

A 58.6% knowledge score indicates limited knowledge in certain critical aspects. Similar findings (57.7% in Knowledge) were reported in Addis Ababa, Ethiopia (33). Our findings revealed that higher education strongly predicted good knowledge. Our study mirrors studies in Kenya and Turkey, emphasizing that literacy is a powerful determinant of AMR-related knowledge (34, 35). Significant district-level disparities in Knowledge may suggest that localized outreach and access to relevant knowledge and extension services are unevenly distributed.

### Attitudes

Nearly all farmers (99.4%) affirmed the importance of seeking professional care when animals are sick. A KAP study of dairy farmers in Turkey reported similar results; 84% of the participants took the advice of the veterinarian before using antibiotics (35). This is a good indication for future interventions. On the other hand, a significant proportion (97.5%) believed antibiotics were necessary in dairy farming and 91.7% supported their use for disease prevention. About 60% believed that antibiotics could increase milk production in cattle. A proportion of 12.2% believed that misusing antibiotics can lead to the emergence of germs that can cause disease in human beings, much lower than the 38.4% reported in North-Western Ethiopia (36).

Worryingly, 38.3% of SHDFs in this study believed that boiling milk could eliminate antibiotic residues, reflecting a critical misconception about the chemical stability of these drugs and the fundamentals of food safety. This belief is particularly concerning, as it may foster a false sense of safety among farmers and consumers, leading to the continued sale and consumption of milk containing harmful residues, especially within informal dairy markets. However, studies have shown that many antibiotics such as tetracyclines and beta-lactams are thermally stable and can persist even after pasteurization or boiling (37). These misconceptions can undermine AMS efforts by perpetuating consumer exposure to residues, limiting public demand for residue-free milk, and weakening compliance with withdrawal periods. Addressing this belief through targeted education campaigns is essential, particularly in regions where informal milk trade is dominant and regulatory frameworks are weak.

Attitudes were strikingly poor, with only 14.4% of respondents expressing positive orientations. Notably, female farmers and older respondents were more likely to hold favorable attitudes. The higher likelihood of favorable attitudes among older farmers and those of female gender may reflect accumulated experiential knowledge and risk aversion tendencies respectively. A combination of social, cultural, and individual experiences may elucidate this trend.

In many rural areas of sub-Saharan Africa, like Zambia, women are more directly involved in fundamental livestock management chores like milking, cleaning, and caring for calves. This means they are more likely to be exposed to disease risks and hygienic practices (33). This repeated involvement may increase awareness of risks and improve attitudes toward infection prevention and responsible use of antibiotics. Additionally, women often take on important roles in making health decisions for the family, which may lead to more careful and preventative approaches to using antibiotics (38, 39).

Interventions should therefore adopt gender-sensitive and age-targeted behavioral strategies, particularly focusing on younger male farmers who dominate production but exhibit weaker attitudes in relation to AMS principles. Given the strong positive orientation toward veterinarian advice, leveraging this trusted relationship could improve farmer attitudes by increasing veterinarian-farmer engagement.

### Practices

Antibiotic use in dairy farming was high in this study, as all the farmers reported using antibiotics in their production systems. A similar trend was also observed in Zimbabwe and Rwanda where extensive use of antibiotics in the dairy sector was documented (12, 40). The excessive use of antibiotics could be linked to farmers' efforts to promote growth, treat various infections such as mastitis, and boost milk production. Other researchers have also observed high rates (over 80%) of antibiotic use in dairy farming (12, 34). The rate of antibiotic use is projected to increase globally (1).

Most farmers (83.3%) reported that they relied on veterinary professionals for diagnosis of disease. Similarly, in Rwanda, a study showed that 73.5% of farmers call a veterinarian when their cattle get sick and in Peru, 95 % reported that antibiotics were prescribed exclusively by a veterinarian (40, 41). A KAP study of dairy farmers in Turkey reported similarly that 84% of the participants took the advice of the veterinarian before using antibiotics (35). However, almost 40% reported self-administration of the drugs, similar to findings in Rwanda (40). Akin to previous findings in Kenya and Ghana most farmers in our study reported obtaining antibiotics from veterinary drug stores (11). The apparent contradiction between high reliance on veterinarians (83.3%) and substantial self-administration (38.6%) reflects a common scenario in LMICs where small scale farmers may initially consult veterinarians for diagnosis but subsequently administer antibiotics independently due to cost constraints, limited follow-up services, or urgency of treatment. Similar patterns have been documented in LMICs, where veterinary advice coexists with informal practices (11).

This practice is often associated with seeking advice and antimicrobial agents from agrovet shops reported in our study that has been documented in similar studies, often resulting in inappropriate drug selection and use (42). Importantly, most agrovet shop attendants are often not trained animal health professionals which further exacerbate inappropriate antimicrobial use practices in livestock systems (11).

A multicountry study that included Zambia reported that economic constraints, especially financial limitations, play a significant role in defining antimicrobial use behaviors, which typically drive farmers to prioritize cost above quality of veterinary care (11).

Similarly, a lack of standard guidelines for antimicrobial use in the animal health sector was noted to be a key challenge to promote and guide rational use in the sector (43). This observed gap was reported to promote the irrational use of drugs. Strengthening access to veterinary services and developing coordinated antimicrobial governance frameworks have been recognized as essential strategies to combat inappropriate antibiotic use in livestock systems (43–45).

These strengthened regulatory frameworks will ensure antibiotic drugs are dispensed with clear, standardized instructions, alongside training agro-veterinary shop attendants, could reinforce good practices at the point of purchase.

The results of the current study show that 71.9% of farmers indicated that they followed the drug withdrawal period, consistent with the findings of Kallu and others (33). The fact that some of the farmers still show noncompliance with antibiotic drug withdrawal periods can lead to the occurrence of ARs in dairy products. In turn, this can stimulate the emergence of new pathogens with AMR genes (46).

Unfortunately, almost a quarter (24.2%) admitted to deviating from prescribed dosage or treatment duration, with 71.3% of these declaring financial constraints as the biggest barrier, while 16.1% said they stopped treatment if the cow appeared healed. In a low-income setting in Peru, 21 % reported that they interrupted the treatment when clinical signs disappeared (41). A study in Sudan documented this same practice, where farmers stated that they often terminate drug administration to animals before completing the antibiotic course to save money by shortening the treatment period (47). These findings point to financial constraints and low risk perception; common barriers documented in similar studies (30, 31). In contrast, Eltayb and others reported that 46.2% of participants said they increased the antibiotic dose and frequency of administration as long as the animals showed no signs of recovery (47). They also reported that participants stopped giving antibiotics if animals appeared to be recovering at some point after using antibiotics. These findings indicate that farmers commonly take the initiative to change antibiotic doses.

The use of antibiotics for disease prevention or growth promotion, reported by 43.3% reflects a prevailing perception of antibiotics as a multipurpose tool, blurring the lines between therapeutic and non-therapeutic uses. An evaluation of cattle farmers' KAP in Sudan, Rwanda and Tanzania also reported this practice in a significant proportion (29, 40, 47).

Of particular concern is that 20% of the participants admitted to selling milk after administration of antibiotics to animals, while 19.7% reported consuming such milk. Similar practices are documented (48). These practices have direct implications for public health and AMR, potentially contributing to low-level antibiotic exposure in consumers, which can drive resistant infections.

The observations made in the domain of practices reinforce that good practices are not solely a function of good knowledge but are deeply embedded in structural and economic limitations.

### Overall KAP

Overall scores were poor, with 75.8% respondents scoring below the threshold of 75%. These findings are consistent with previous studies in sub-Saharan Africa which reported low or poor KAP scores among dairy farmers (13, 33, 40, 49).

Various studies have found a strong relationship between KAP and other demographic factors including education level and place of residence (12, 13, 40, 47). In the current study, a statistically significant relationship between KAP outcome and these two variables (education and geographic location) was observed. Education emerged as a key determinant of good KAP. Participants with secondary or tertiary education were significantly more likely to demonstrate good KAP than those with primary or no education. These findings reinforce the role of formal education in enhancing risk awareness, critical thinking, and adherence to public health recommendations.

Geographical location was another significant predictor. Farmers from Monze district exhibited notably higher KAP scores compared to the other districts. Comparatively, Chilanga district of Lusaka province had the highest KAP scores and Monze district of Southern province. Both these districts are distinctly located in a peri-urban setup in comparison to the other districts in their respective provinces. This may reflect differences in access to veterinary education and services and warrants further investigations. The observed geographical disparities in KAP outcomes also suggests that future interventions must be context-specific and evidence-based to address relevant gaps. This study did not collect data on disease prevalence across districts. However, differences in disease burden (e.g., mastitis prevalence) may partly explain variations in antimicrobial use practices and should be explored in future research.

Training also significantly influenced KAP outcomes. Participants who had received training on prudent antibiotic use and animal health management were more likely to have good KAP. This finding supports the evidence that farmer education and training programmes can positively shift attitudes and behaviors Other studies have already established that literacy levels on dairy farming and antibiotic use and their effects are an essential factor for improving overall KAP among dairy farmers (12, 35, 36).

### Correlation across domains

The Spearman correlation analysis revealed positive associations between knowledge, attitudes, and practices. The strongest correlation was observed between knowledge and practices, followed by attitudes and practices, and knowledge and attitudes. These findings suggest that while improving knowledge may contribute to better practices, the relationship is modest and influenced by multiple external factors. Attitudes and practices on antibiotic use are influenced by several individual levels and environmental factors, which cannot be addressed by improving knowledge alone, such as those driven by economic motives (31). For example, a farmer may know about the risks of not observing withdrawal periods after the administration of antibiotics to most of his cows but may not observe the required waiting time due to fear of encountering profit losses as evidenced by the 20% of participants who admitted that they still sold milk after administering antibiotic drugs. Empirical research suggests that while knowledge is a necessary foundation, behavior change requires enabling environments, reinforcement, and community engagement (50). For example, more stringent enforcement of regulations and a robust national veterinary drug residue monitoring system will also be necessary to encourage compliance and protect public health.

### Alignment of KAP findings with the Zambia national one health strategic plan (2022–2026)

The findings of this study were interpreted in relation to the Zambia National One Health Strategic Plan (2022–2026), with particular emphasis on antimicrobial resistance (AMR), food safety, and multisectoral system strengthening (51).

In relation to Strategic Objective 1 (Strengthening One Health Governance and Coordination), the observed variability in knowledge and practices across districts points to uneven implementation of risk communication and stakeholder engagement strategies. Re-emphasizing the need for targeted, context-specific AMS interventions.

With respect to Strategic Objective 2 (enhancing surveillance systems for priority zoonotic diseases and AMR), the persistence of risky practices, including non-adherence to withdrawal periods and informal antibiotic use indicates that surveillance strengthening cannot be limited to laboratory and institutional capacity but must incorporate behavioral compliance at the production level. For example limiting the purchase of antibiotics to prescription only.

In relation to Strategic Objective 3 (strengthening prevention and control of public health threats), while some level of awareness exists, it does not consistently translate into safe practices, suggesting that existing strategies may be highly focused on information dissemination rather than sustained behavior change. There is therefore need for integrated interventions that combine education, regulation, and economic incentives to influence farmer decision-making.

Strategic Objective 4 (workforce development and capacity building) requires a more nuanced interpretation in light of the findings. The observed association between training and improved KAP outcomes confirms the importance of capacity development. Importantly, workforce development within the One Health framework extends beyond farmer training to include the availability, accessibility, and functionality of veterinary and extension services, as well as the capacity of front-line personnel to deliver continuous, context-specific support.

Overall, the alignment of KAP findings with the Zambia National One Health Strategic Plan highlights the need for a shift from awareness-driven approaches toward integrated, behavior-centered, and system-supported interventions. Without addressing both individual-level behaviors and structural constraints, the impact of One Health strategies on AMS and food safety is likely to remain limited.

## Conclusion

This study provides one of the first systematic assessments of KAP regarding AMU and AMR among SHDFs in Zambia. This study demonstrates that while knowledge and certain practices regarding antimicrobial use were acceptable among SHDFs in Zambia, attitudes remain a critical weak point, undermining overall stewardship efforts. Financial constraints further limit prudent AMU. This indicates that improving AMU is not merely a matter of awareness, but also of accessibility and affordability. One such intervention would be the introduction of subsdies to offset the cost of antibiotics to enhance farmer compliance with treatment time. Bridging the gap between knowledge, attitudes, and practices will require integrated interventions that are educational, economic, and policy-driven. Empowering farmers through training, improving access to affordable veterinary services, and embedding AMU messaging into community-level dialogues is recommended. In conclusion, AMS in the dairy sector must be seen as both a scientific and social endeavor. By aligning knowledge with supportive systems and nurturing positive attitudes, Zambia and other LMICs can strengthen food safety, public health, and contribute meaningfully to the global fight against AMR.

### Study limitations and further research

A strength of this study was the use of a large, geographically diverse sample and statistical analysis, including multivariable logistic regression models and correlation testing. However, study limitations included:

#### Self-reported data and social desirability bias

The study relied heavily on self-reported responses from surveys. This approach may have introduced interviewer influence and social desirability bias, whereby participants provided responses that align with perceived expectations or socially acceptable behaviors. Participants may have underreported or misrepresented their behaviors. This social desirability bias could have led to an underestimation of high-risk behaviors. To minimize this effect, participants were assured of anonymity and confidentiality. However, future research could incorporate direct observations and/or ethnographic methods.

#### Cross-sectional study design

This study employed a cross-sectional design, capturing data at a single point in time. While this approach provided a snapshot of community KAPs, it did not assess changes over time. Future studies should incorporate longitudinal designs to track how knowledge, attitudes, and practices evolve, particularly in response to interventions.

#### Socio-economic variables

This study did not include some socio-economic variables such as income level, herd size, farming experience, and access to veterinary services, which have been used in similar studies and may influence antimicrobial use behaviors (11, 31).

#### Sampling bias

Participants were recruited using convenience sampling at the MCCs, which may have excluded farmers operating outside cooperative structures or informal milk markets. These farmers may have lower access to veterinary services and potentially poorer AMU practices. As such, the study findings may overestimate KAP levels among the broader population of small scale farmers.

## Data Availability

The raw data supporting the conclusions of this article will be made available by the authors, without undue reservation.
