# Correction: Knowledge, attitudes and practices of smallholder dairy farmers on antimicrobial use in selected districts of Zambia: implications for antimicrobial stewardship

**DOI:** 10.3389/fvets.2026.1926436

**Published:** 2026-08-28

**Authors:** Lweendo Hachamba, Ethel M'kandawire, John Bwalya Muma, Flavien Bumbangi, Noela Lukwesa, Mapeesho Kamayani, Merning Mwenifumbo, Mette Helen Bjorge Müller

**Affiliations:** 1 Department of Environmental Health, School of Medicine, Eden University, Barlastone Park, Lusaka, Zambia; 2 Department of Disease Control, School of Veterinary Medicine, University of Zambia, Lusaka, Zambia; 3 School of Medicine, Eden University, Barlastone Park, Lusaka, Zambia; 4 Churches Health Association of Zambia, Lusaka, Zambia; 5 Department of Veterinary Public Health and Epidemiology, Lilongwe University of Agriculture and Natural Resources, Lilongwe, Malawi; 6 Faculty of Veterinary Medicine, Norwegian University of Life Sciences, NMBU, Ås, Norway

**Keywords:** antibiotic resistance, antibiotic stewardship, antibiotic use, antibiotics, attitudes, knowledge, practices, smallholder dairy farmers

In the published article, the figures were listed in an incorrect order.

Figure 1 was listed as Figure 5, Figure 2 as Figure 1, Figure 3 as Figure 2, Figure 4 as Figure 3, Figure 5 as Figure 4. The correct figure order and their caption appear below.

**Figure 1:**
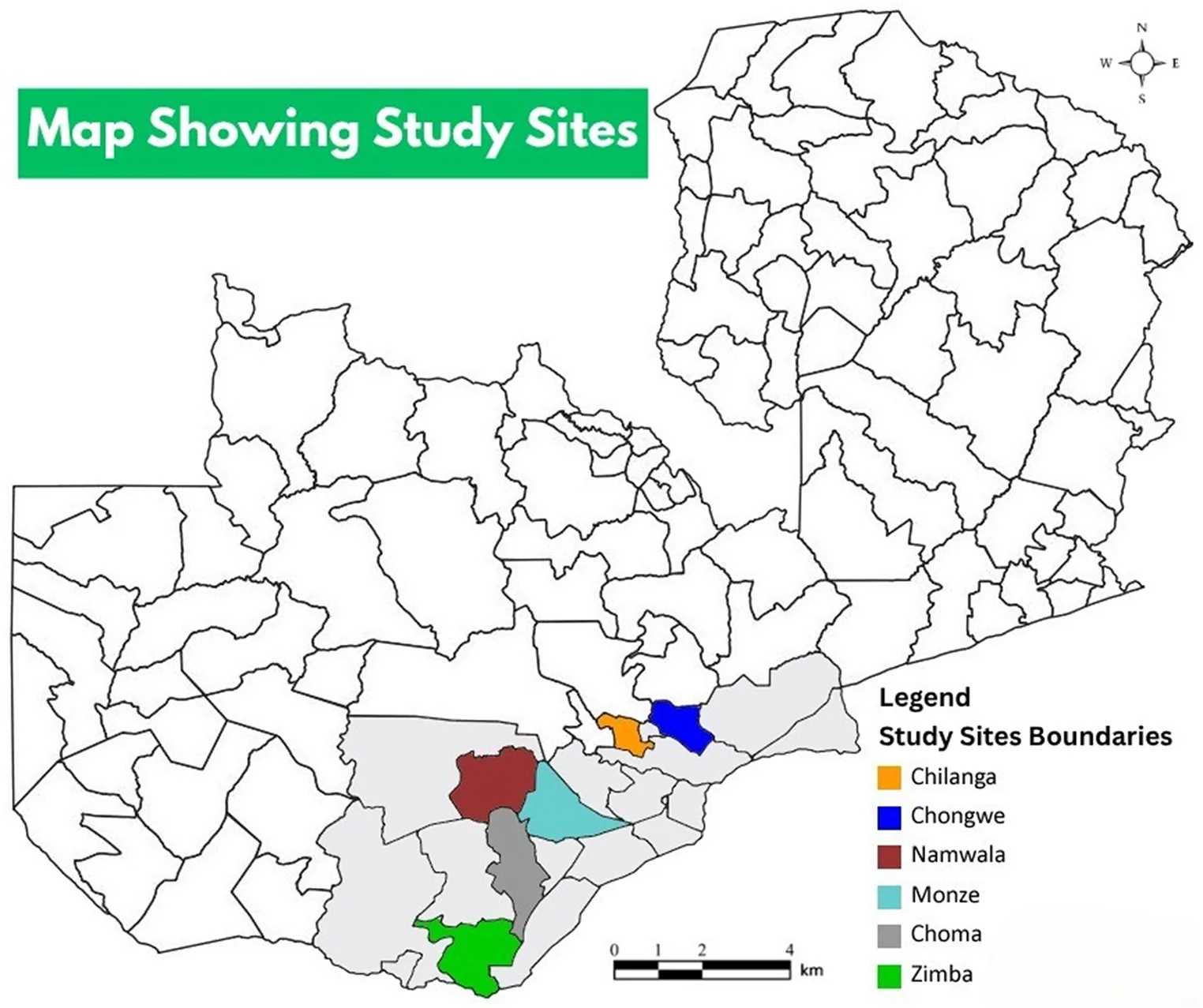
Map of study sites.

**Figure 2:**
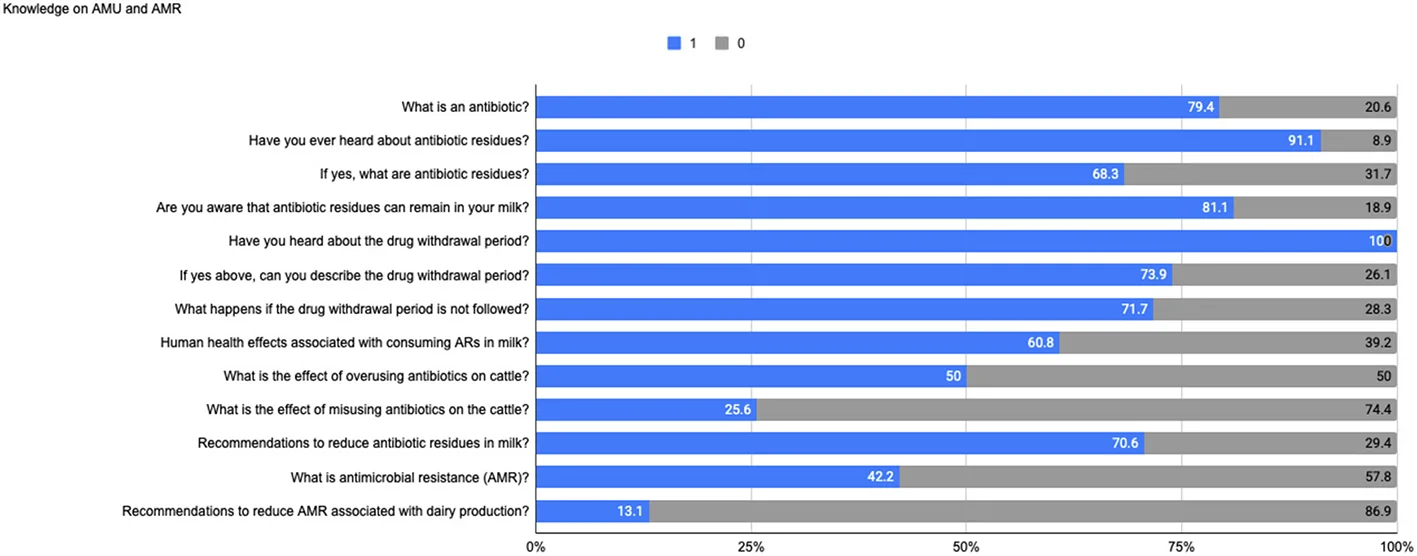
Knowledge on AMU and AMR.

**Figure 3:**
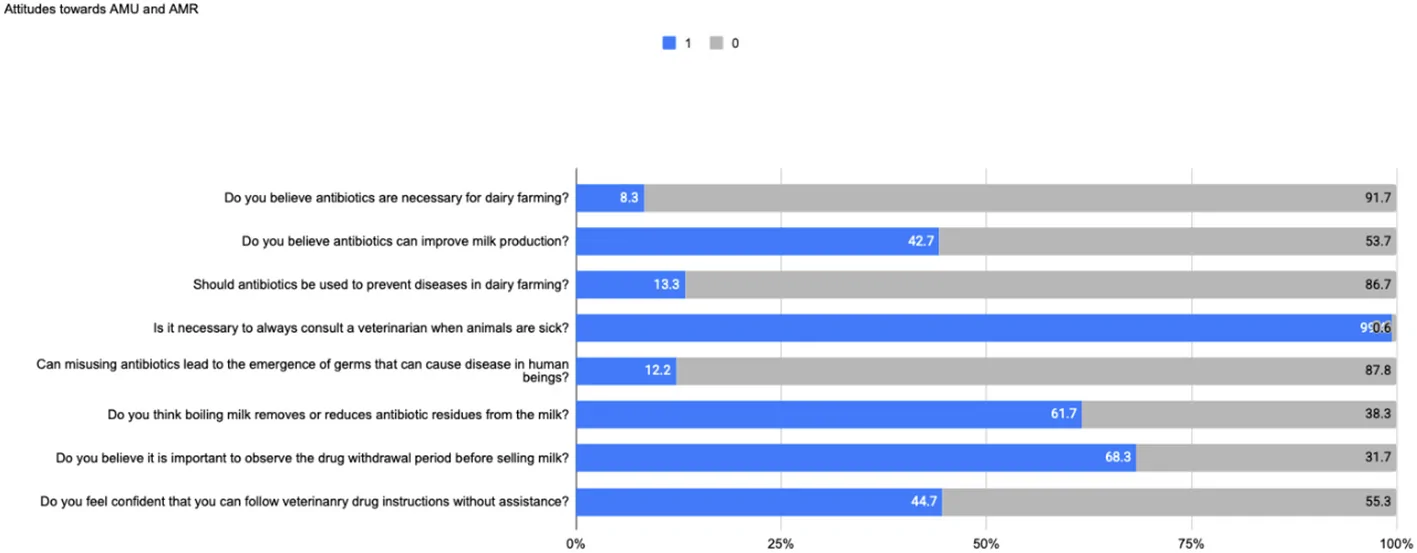
Attitudes towards AMU and AMR.

**Figure 4:**
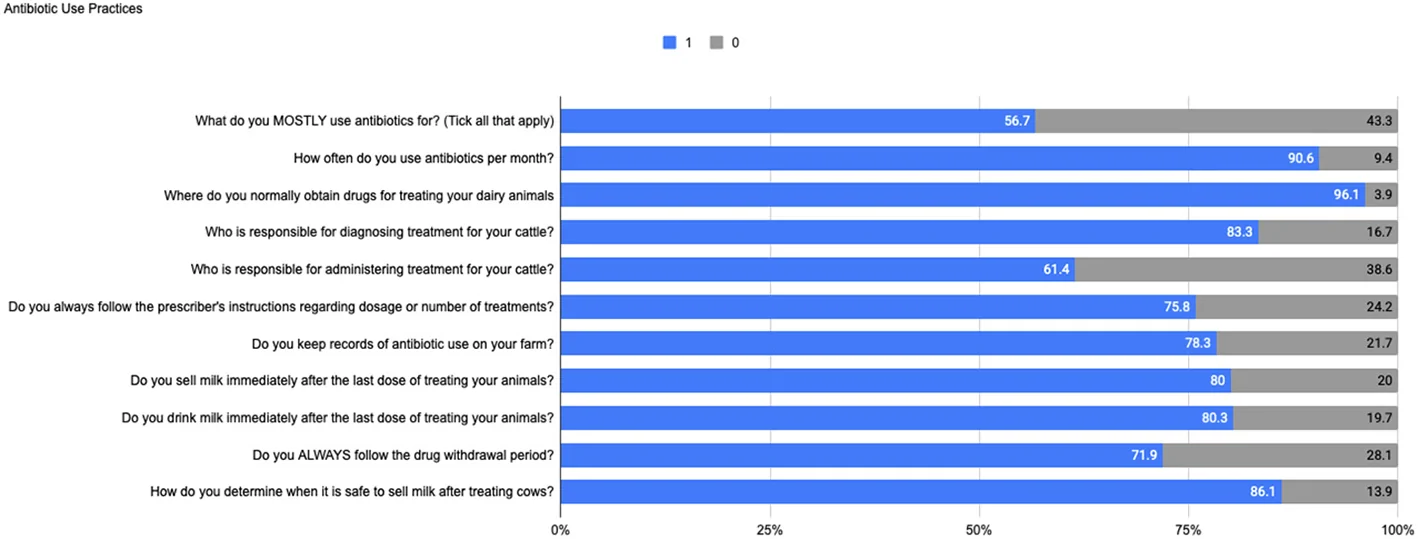
Antibiotic use practices.

**Figure 5:**
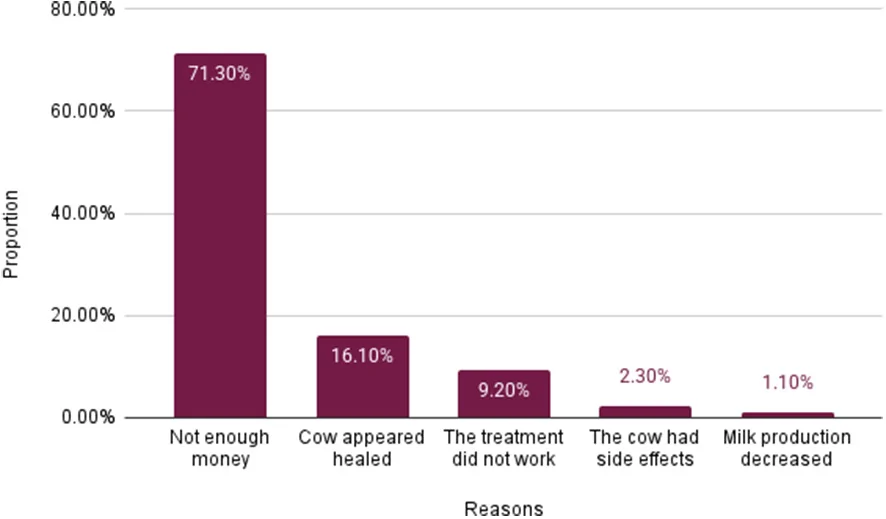
Reasons for not following prescription.

The original version of this article has been updated.

