# Supplementary material for: Knowledge, attitudes and practices of smallholder dairy farmers on antimicrobial use in selected districts of Zambia: implications for antimicrobial stewardship
Source: Front Vet Sci. 2026 Jun 11;13:1763931. doi: 10.3389/fvets.2026.1763931 (PMC13295105; doi:10.3389/fvets.2026.1763931)
Supplement: Supplementary file 1 [file Table_1.DOCX]

**Table 1. Knowledge Scores across Demographic Variables**

| **Variable** | **N** | **Knowledge** | | ***p*-Value** |
| --- | --- | --- | --- | --- |
|  |  | **Good** | **Poor** |  |
| **Gender** | | | | |
| Male | 303 | 174(57.4%) | 129(42.6%) | 0.292 |
| Female | 57 | 37(65%) | 20(35%) |  |
| **Age Range** |  |  |  |  |
| 18-24 | 17 | 9 (53%) | 8 (47%) | 0.022 |
| 25 – 34 | 82 | 43(52.4%) | 39 (47.6%) |  |
| 35 – 44 | 113 | 57(50.4%) | 56(49.6%) |  |
| 45 – 54 | 83 | 59(71.1%) | 24(28.9%) |  |
| 55 – 64 | 37 | 27(73%) | 10(27%) |  |
| 65 + | 28 | 16(57.1%) | 12(42.8%) |  |
| **Formal Education** | | | | |
| No Education | 12 | 5(41.7%) | 7(58.3%) | <0.001 |
| Primary | 143 | 68(47.6%) | 75(52.4%) |  |
| Secondary | 110 | 70(63.6%) | 40(36.4%) |  |
| Tertiary | 95 | 68(71.6%) | 27(28.4%) |  |
| **District** | | | | |
| Choma | 65 | 30(46.2%) | 35(53.8%) | <0.001 |
| Chongwe | 74 | 20(27%) | 54(73%) |  |
| Monze | 85 | 78(91.8%) | 7(8.2%) |  |
| Namwala | 50 | 32(64%) | 18(36%) |  |
| Zimba | 50 | 42(84%) | 8(16%) |  |
| Chilanga | 36 | 9(25%) | 27(75%) |  |
