# Supplementary material for: Knowledge, attitudes and practices of smallholder dairy farmers on antimicrobial use in selected districts of Zambia: implications for antimicrobial stewardship
Source: Front Vet Sci. 2026 Jun 11;13:1763931. doi: 10.3389/fvets.2026.1763931 (PMC13295105; doi:10.3389/fvets.2026.1763931)
Supplement: Supplementary file 2 [file Table_2.DOCX]

**Table 2. Attitude Scores across Demographic Variables**

| **Variable** | **N** | **Attitude** | | ***p*-Value** |
| --- | --- | --- | --- | --- |
|  |  | **Good** | **Poor** |  |
| **Gender** | | | | |
| Male | 303 | 37(12.2%) | 266(87.8%) | 0.005 |
| Female | 57 | 15(26.3%) | 42(73.7%) |  |
| **Age Range** | | | | |
| 18-24 | 17 | 1(5.9%) | 16(94.1%) | 0.012 |
| 25 – 34 | 82 | 5(6.1%) | 77(93.9%) |  |
| 35 – 44 | 113 | 18(15.9%) | 95(84.1%) |  |
| 45 – 54 | 83 | 11(13.3%) | 72(86.7%) |  |
| 55 – 64 | 37 | 8(21.6%) | 29(78.4%) |  |
| 65 + | 28 | 9(32.1%) | 23(67.9%) |  |
| **Formal Education** | | | | |
| No Education | 12 | 1(8.3%) | 11(91.7%) | <0.001 |
| Primary | 143 | 12(8.4%) | 131(91.6%) |  |
| Secondary | 110 | 13(11.8%) | 97(88.2%) |  |
| Tertiary | 95 | 26(27.4%) | 69(72.6%) |  |
| **District** | | | | |
| Choma | 65 | 7(10.8%) | 58(89.2%) | 0.006 |
| Chongwe | 74 | 5(6.8%) | 69(93.2%) |  |
| Monze | 85 | 14(16.5%) | 71(83.5%) |  |
| Namwala | 50 | 5(10%) | 45(90%) |  |
| Zimba | 50 | 9(18%) | 41(82%) |  |
| Chilanga | 36 | 12(33%) | 24(67%) |  |
