# Supplementary material for: Knowledge, attitudes and practices of smallholder dairy farmers on antimicrobial use in selected districts of Zambia: implications for antimicrobial stewardship
Source: Front Vet Sci. 2026 Jun 11;13:1763931. doi: 10.3389/fvets.2026.1763931 (PMC13295105; doi:10.3389/fvets.2026.1763931)
Supplement: Supplementary file 3 [file Table_3.DOCX]

**Table 3. Practice Scores across Demographic Variables**

| **Variable** | **N** | **Practices** | | ***p*-Value** |
| --- | --- | --- | --- | --- |
|  |  | **Good** | **Poor** |  |
| **Gender** | | | | |
| Male | 303 | 191(63%) | 112(37%) | 0.463 |
| Female | 57 | 33(57.9%) | 24(42.1%) |  |
| **Age Range** | | | | |
| 18-24 | 17 | 9(52.9%) | 8(47.1%) | 0.238 |
| 25 – 34 | 82 | 50(61%) | 32(39%) |  |
| 35 – 44 | 113 | 62(54.9%) | 51(45.1%) |  |
| 45 – 54 | 83 | 57(68.7%) | 26(31.3%) |  |
| 55 – 64 | 37 | 26(70.3%) | 11(29.7%) |  |
| 65 + | 28 | 20(71.4%) | 8(28.6%) |  |
| **Education** | | | | |
| No Education | 12 | 5(41.7%) | 7(58.3%) | 0.011 |
| Primary | 143 | 100(69.9%) | 43(30.1%) |  |
| Secondary | 110 | 57(51.8%) | 53(48.2%) |  |
| Tertiary | 95 | 62(65.3%) | 33(34.7%) |  |
| **District** | | | | |
| Choma | 65 | 58(89.2%) | 7(10.8%) | <0.001 |
| Chongwe | 74 | 24(32.4%) | 50(67.6%) |  |
| Monze | 85 | 79(92.9%) | 6(7.1%) |  |
| Namwala | 50 | 7(14%) | 43(86%) |  |
| Zimba | 50 | 35(70%) | 15(30%) |  |
| Chilanga | 36 | 21(58.3%) | 15(41.7%) |  |
