# Supplementary material for: Knowledge, attitudes and practices of smallholder dairy farmers on antimicrobial use in selected districts of Zambia: implications for antimicrobial stewardship
Source: Front Vet Sci. 2026 Jun 11;13:1763931. doi: 10.3389/fvets.2026.1763931 (PMC13295105; doi:10.3389/fvets.2026.1763931)
Supplement: Supplementary file 4 [file Table_4.DOCX]

**Table 4. Association between Good KAP and Training**

| **X^2^** | **OR** | **95%CI** | ***p*-Value** |
| --- | --- | --- | --- |
| 5.65 | 0.556 | 0.342-0.905 | 0.017 |
